# Development of an All-in-One Inducible Lentiviral Vector for Gene Specific Analysis of Reprogramming

**DOI:** 10.1371/journal.pone.0041007

**Published:** 2012-07-18

**Authors:** Tomoyuki Yamaguchi, Sanae Hamanaka, Akihide Kamiya, Motohito Okabe, Mami Kawarai, Yukiko Wakiyama, Ayumi Umino, Tomonari Hayama, Hideyuki Sato, Youn-Su Lee, Megumi Kato-Itoh, Hideki Masaki, Toshihiro Kobayashi, Satoshi Yamazaki, Hiromitsu Nakauchi

**Affiliations:** 1 Japan Science Technology Agency, ERATO, Nakauchi Stem Cell and Organ Regeneration Project, Tokyo, Japan; 2 Division of Stem Cell Therapy, Center for Stem Cell Biology and Regenerative Medicine, Institute of Medical Science, University of Tokyo, Tokyo, Japan; Montana State University, United States of America

## Abstract

Fair comparison of reprogramming efficiencies and *in vitro* differentiation capabilities among induced pluripotent stem cell (iPSC) lines has been hampered by the cellular and genetic heterogeneity of de novo infected somatic cells. In order to address this problem, we constructed a single cassette all-in-one inducible lentiviral vector (Ai-LV) for the expression of three reprogramming factors (*Oct3/4*, *Klf4* and *Sox2*). To obtain multiple types of somatic cells having the same genetic background, we generated reprogrammable chimeric mice using iPSCs derived from Ai-LV infected somatic cells. Then, hepatic cells, hematopoietic cells and fibroblasts were isolated at different developmental stages from the chimeric mice, and reprogrammed again to generate 2nd iPSCs. The results revealed that somatic cells, especially fetal hepatoblasts were reprogrammed 1200 times more efficiently than adult hepatocytes with maximum reprogramming efficiency reaching 12.5%. However, we found that forced expression of *c-Myc* compensated for the reduced reprogramming efficiency in aged somatic cells without affecting cell proliferation. All these findings suggest that the Ai-LV system enables us to generate a panel of iPSC clones derived from various tissues with the same genetic background, and thus provides an invaluable tool for iPSC research.

## Introduction

Induced pluripotent stem cells (iPSCs) are artificial pluripotent stem cells originally generated from mouse somatic cells in 2006 [1] and from human somatic in 2007 [2], [3] by the enforced expression of four transcription factors (*Oct 4*, *Sox2*, *c-Myc*, and *Klf4*); genes that are expressed in embryonic stem cells (ESCs). IPSCs are alkaline phosphatase positive and morphologically similar to ESCs and are likewise capable of differentiating into cell types representative of all three germ layers: ectoderm, endoderm, and mesoderm. Moreover, gene expression profiles, chromatin methylation patterns and doubling time of iPSCs closely resemble those of ESCs, but the full extent of their relationship is still being evaluated. Besides the ethical issues surrounding the use of human embryos, there is a technical problem of graft-versus-host disease associated with allogeneic stem cell transplantation. However, these problems may be solved using autologous iPSCs and this is an important advantage of iPSCs relative to ESCs in the development of iPSCs-based therapies. On the other hand, detailed mechanisms of reprogramming or differentiation capacity of iPSCs are not clearly understood and elucidation of these subjects is important to both basic and translational research. A number of methods for generating iPSCs have been reported. These include DNA [4], [5], [6], [7], RNA [8], [9] and protein [10] transfection as well as the viral delivery systems including retrovirus [1], lentivirus [11], [12], adenovirus [13] and sendaivirus [14], [15]. Because iPSCs are generally produced by the overexpression of three or four transcriptional factors, the generated clones display genetic heterogeneity and this is an obstacle to understanding their mechanisms of reprogramming and phenotypes. In light of this problem, several groups have reported a reprogrammable mouse system using transgenic mice carrying two tetracycline (tet) inducible vectors consisting of a tet responsive element (TRE) driven TA peptide connected to four reprogramming factors (*Oct3/4*, *Klf4*, *Sox2* and *c-Myc*) and reverse tet transactivator (rtTA) on the ROSA locus [11], [12], [16], [17]. Because somatic cells isolated from reprogrammable mice or their iPSCs carry the same genetic background, a fair comparison of reprogramming efficiency or phenotype of iPSCs are possible.

One of the proto-oncogenes, *Myc* is known to interact with proteins essential for transcriptional regulation such as transformation/transcription domain-associated protein (TRRAP) or histone acetyltransferases (HAT), and this is considered to be important for multiple functions of *Myc,* like regulation of cell cycle, metabolism, differentiation, transformation and apoptosis [18], [19]. *Myc* also plays a crucial role in reprogramming, since its absence significantly lowered reprogramming efficiency [20]. It has also been reported that the efficiency of germline transmission of iPSCs largely depends on *Myc* transgenes [21], [22]. However, these results were obtained using materials that were not genetically identical.

To circumvent this problem, we constructed a single cassette all-in-one inducible lentiviral vector (Ai-LV) for expression of three reprogramming genes (*Oct4*, *Sox2* and *Klf4*) self-cleaving 2A peptides and a tetracycline inducible expression module. It should be noted that somatic cells of different types are available from reprogrammable chimeric mice using iPSCs derived from Ai-LV infected somatic cells and the function of *c-Myc* on reprogramming can be easily analyzed by the additional expression of *c-Myc*. Moreover, because we used a single cassette, this system could easily create reprogrammable animals other than mouse; which could not be accomplished with the previous system.

## Results

### Generation of Primary iPSCs by All-in-one Inducible Lentiviral Vector (Ai-LV)

**Figure 1:**
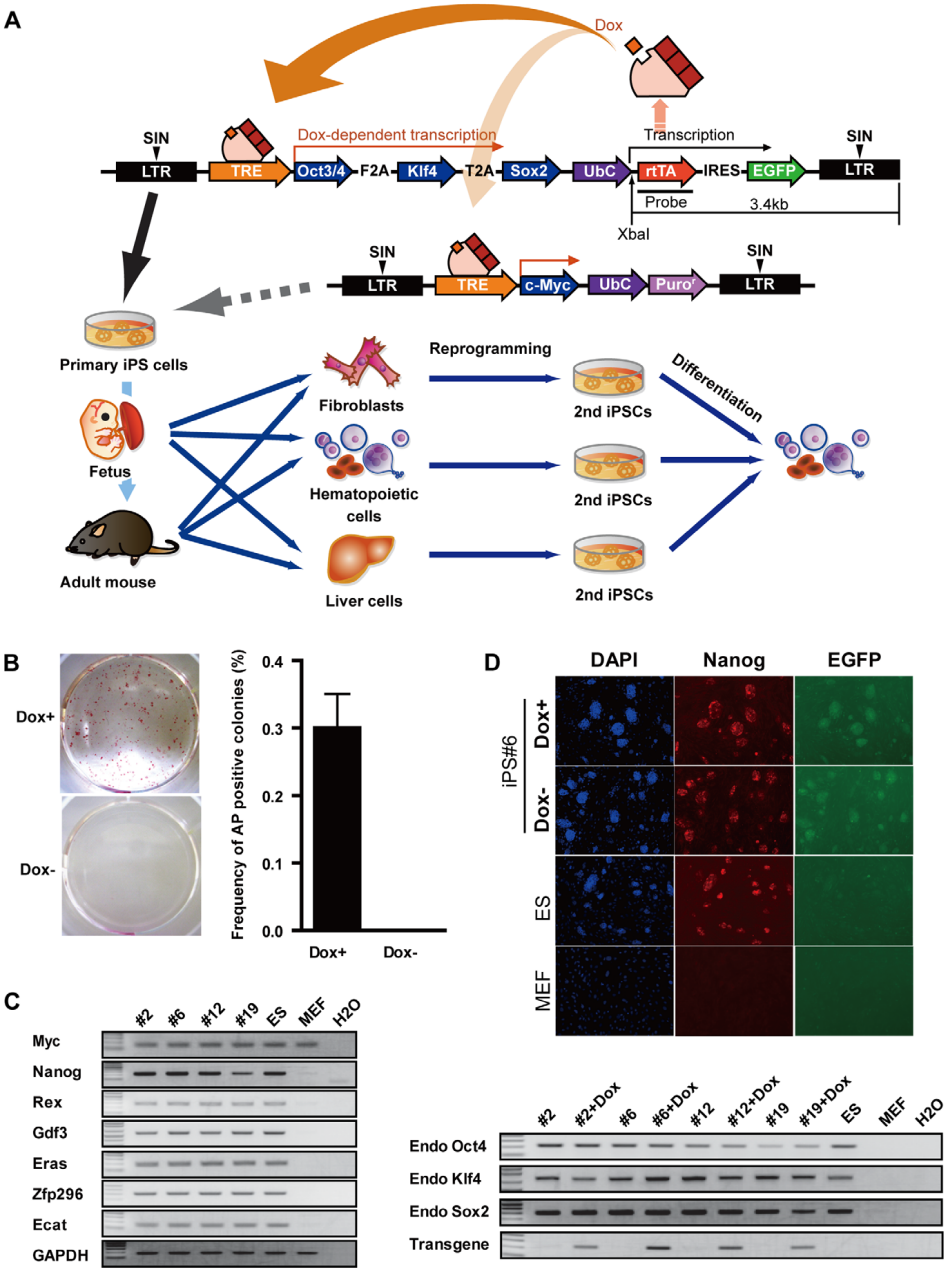
Construction of Dox inducible reprogramming system. (A) Schematic diagram of Dox inducible system for expression of reprogramming factors. (B) Alkaline phosphatase (AP) staining of iPS colonies derived from Ai-LV transduced MEFs (left panel). Efficiency of AP positive colonies (right panel). Efficiency of AP positive colonies calculated by dividing infected cell number by the number of AP positive colonies. (B) RT-PCR analysis of endogenous pluripotent marker genes, with or without Dox in the culture. (C) Immunofluorescence staining for *Nanog* in iPS clone#6, with or without Dox in the culture.

In order to generate a reprogrammable mouse strain, we constructed a Doxycyclin (Dox) dependent inducible lentiviral vector; encoding for tet-responsive element (TRE) regulation of murine versions of three reprogramming factors (Oct4, Klf4 and Sox2) and human ubiquitin C (ubc) promoter-driven reverse tet-transactivator (rtTA) and Enhanced Green Fluorescent Protein (EGFP) connected by an internal ribosomal entry site (IRES) (Fig. 1A). Under the control of a Ubc promoter, rtTA ubiquitously expressed in mouse tissues where it binds to TRE in the presence of Dox and initiates transcription of reprogramming factors. Ai-LV induced iPSCs can be used to generate chimeric mice, from which genetically identical tissues (e.g., fibroblast, hematopoietic cells and hepatic cells) can be isolated at different developmental stages. These tissues can be re-reprogrammed into iPSCs by the addition of Dox in the culture medium for analysis of their reprogramming potential. Moreover, it is possible to measure the differentiation capacity of the re-reprogrammed iPSCs *in vitro*. To further analyze the function of *Myc* during reprogramming, iPSCs generated by Ai-LV were infected with an additional inducible vector carrying myc for re-reprogramming, as described in Fig. 1A.

To generate reprogrammable chimeric mice, we infected mouse embryonic fibroblasts with Ai-LV and cultured with Dox-containing medium. Morphologically ES-like colonies appeared after six to eight days of infection, expressed EGFP and were of typical dome shape. Alkaline phosphatase (AP) staining revealed that all colonies were pluripotent and the number of AP^+^ colonies were 51 at a multiplicity of infection (m.o.i.) of 0.4, 127 at 0.8 and 209 at 1.6, and the efficiency of reprogramming was 0.14% (Fig. 1B). On the other hand, no colonies appeared in Ai-LV infected cells cultured without Dox. Several iPS colonies were isolated and examined for the expression profiles of pluripotent marker genes including *c-Myc*, *Nanog*, *Rex*, *Gdf3*, *Eras*, *Zfp296*, *Ecat*, endogenous *Oct4*, endogenous *Klf4* and endogenous *Sox2* by RT-PCR. To detect transgene expression, we designed the primer to amplify the sequence between the T2A and *Klf4* sequence. As shown in Figure 1C, the pluripotent marker genes were expressed at quantities comparable to those in C57Bl/6 mouse ES cells (B6 ES) and the expression of transgene was detected only in Dox-treated iPSCs. This indicates that iPSCs generated by Ai-LV were completely reprogrammed and the expression from the lentiviral vector was tightly controlled by a TRE. Pluripotency of iPSCs was further confirmed by continuous expression of *Nanog* in both cases with or without Dox (Fig. 1D). To ask whether these clones are capable of re-reprogramming by adding Dox, we performed re-reprogramming of *in vitro* differentiated iPS clones (removal of MEF and Lif for two weeks) and revealed re-reprogramming of all clones (Fig. S1A).

Southern blot analysis revealed that proviral copy numbers are one or two, indicating that one copy of Ai-LV is enough for induction of iPSCs (Fig. S1B).

These results indicate that iPSCs generated by Ai-LV were reprogrammed into a pluripotent state and transgene expression was tightly controlled by a tetracycline inducible expression module. Moreover the pluripotent states of iPSCs generated by Ai-LV were kept, regardless of transgene expression. Because the iPSCs#6 clone carries only one proviral copy and exhibits the highest levels of transgene expression among the four clones, this particular clone was chosen for the generation of chimeric mice. Before attempting to generate chimeric mice, we evaluated iPSCs #6 clone karyotypes; they were normal (40XY; Fig. S1C).

We also tried to generate human iPSCs (hiPSCs) by infection of 3×10^4^ human neonatal dermal fibroblasts with Ai-LV encoding human version of three reprogramming factors (Oct4, Klf4 and Sox2) with or without human *c-MYC* encoding lentiviral vector; however we could only generate two hiPSC colonies when the *c-Myc* vector was infected. Then, we performed *in vitro* differentiation of hiPSCs and observed only iPS colonies in Dox-containing culture (Fig. S1D), indicating that the inducible system described here also works in human somatic cells.

### Phenotype of Secondary iPSCs (2nd miPSCs)

**Figure 2:**
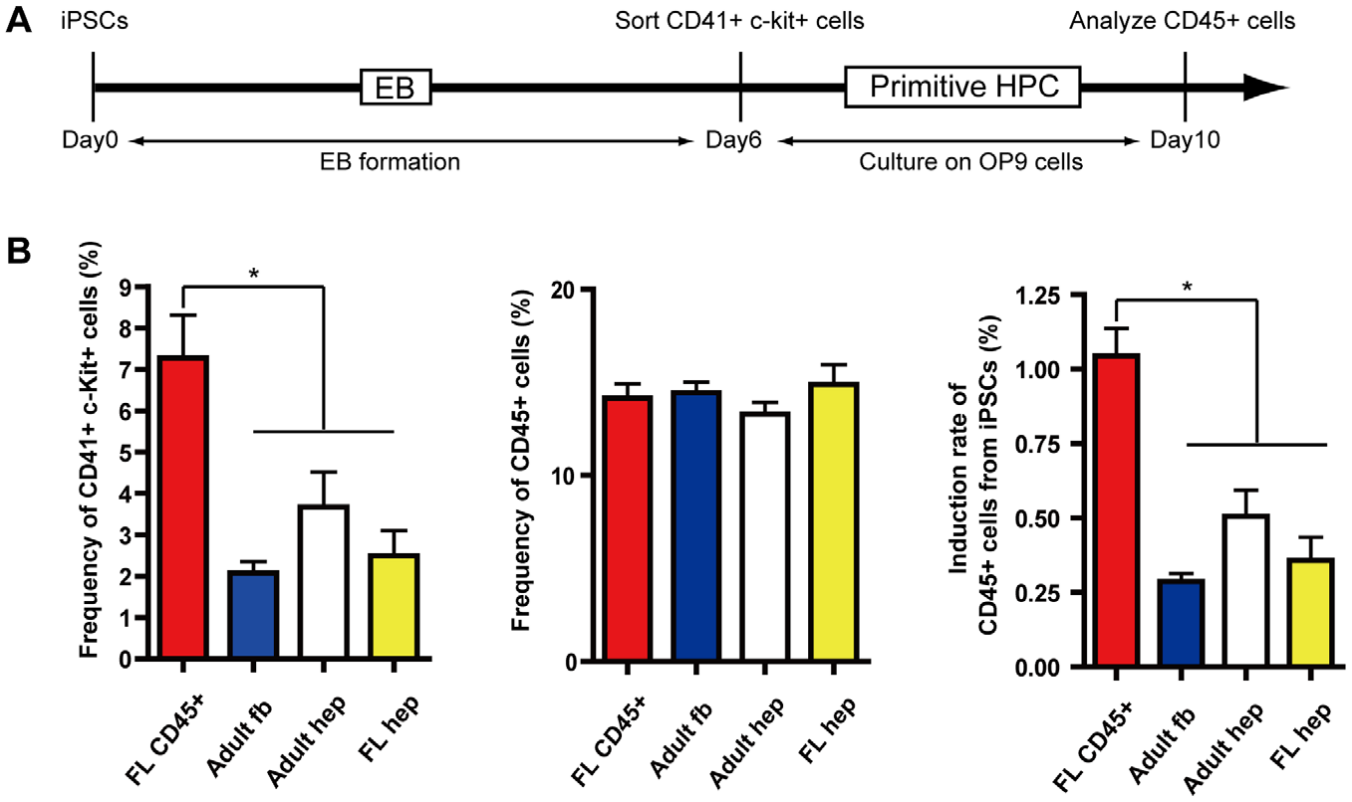
Differentiation capacity of iPSCs to hematopoietic cells. (A) Protocol for in vitro differentiation of iPSCs to hematopoietic cells. (B) Frequency of CD41^+^, c-Kit^+^ primitive hematopoietic progenitor in dissociated EB cells at day six. *p<0.05 (left). Frequency of CD45^+^ hematopoietic cells in sorted CD41^+^, c-Kit^+^ cells cultured on OP9 feeder layer for four days (middle). Total induction rate of CD45^+^ hematopoietic cells from iPSCs were analyzed. *p<0.05 (right).

Previous reports showed that iPSCs derived from murine tissues possessed residual DNA methylation signatures characteristic of their somatic tissue of origin, which tends to differentiate to lineages of the donor cell [23], [24], [25]. To analyze whether the miPSCs re-reprogrammed from chimeric mice (2nd miPSCs) posses this phenotype, we generated 2nd miPSCs from E13.5 fetal liver CD45^+^ hematopoietic cells (FL CD45), adult dermal fibroblasts (Adult fb), adult hepatocytes (Adult hep) and E13.5 fetal hepatoblasts (Fetal hep) and compared their efficiency of induction to hematopoietic cells by *in vitro* differentiation assay (Fig. 2A). As shown in Fig. 2B, differentiation efficiency of each tissue type to CD41^+^c-kit^+^ primitive hematopoietic progenitor cells (primitive HPC) was 7.3%(FL CD45), 2.1% (Adult fb), 3.7% (Adult hep) and 2.0% (Fetal hep). Although no significant differences were observed in the efficiency of differentiation from CD41^+^c-kit^+^ primitive HPC to CD45+ hematopoietic cells (14.2%, 14.4%, 13.3% and 14.9%, respectively) after 4-day culture on OP9 stromal cells, overall differentiation capacity of 2nd miPSCs derived from FL CD45 to CD45^+^ hematopoietic cells was over 2 folds higher than iPSCs derived from other tissues with a statistically significant difference (Fig. 2B). These results observed for our system coincide with previously described epigenetic memories in the 2nd miPSCs [25].

### Analysis of Reprogramming Efficiency

**Figure 3:**
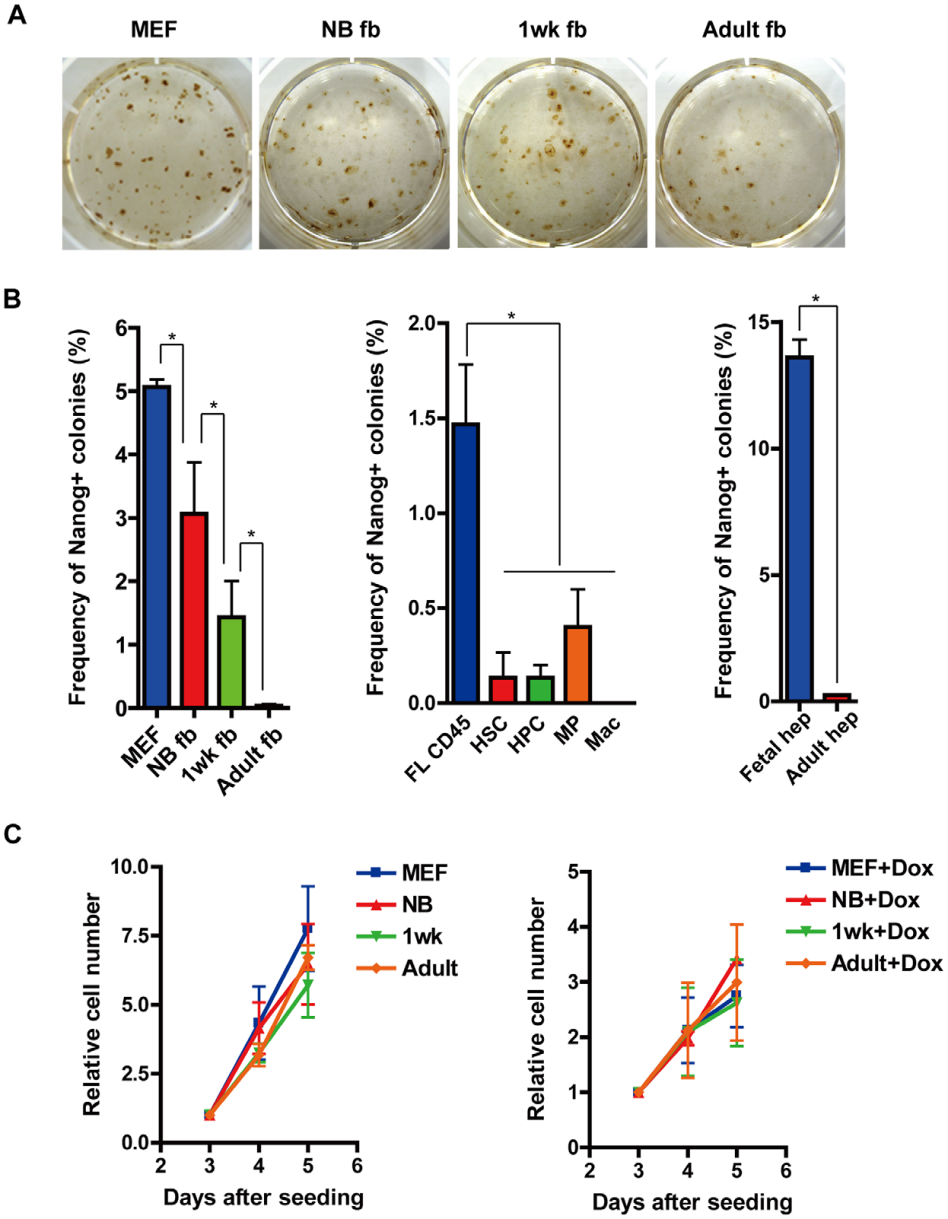
Reprogramming efficiency from somatic cells at different developmental stages. (A) *Nanog* immunostaining of 2^nd^ iPS colonies derived from MEF (2000 cells), NB fb (2000 cells), 1wk fb (5000 cells) and Adult fb (10000 cells). Each cell were seeded on a feeder layer and cultured in the presence of Dox for two weeks. (B) Reprogramming efficiency of fibroblasts (MEF, NB fb, 1wk fb and Adult fb) *p<0.05 (left panel), hematopoietic cells (FL CD45, HSC, HPC, MP and Mac) *p<0.05 (middle panel) and liver cells (Fetal hep and Adult hep) *p<0.01 (right panel) were analyzed by dividing seeded cell number by the number of *Nanog* positive colonies. (C) Cell proliferation rate of fibroblasts (MEF, NB fb, 1wk fb and Adult fb) at three, four and five days after seeded in the absence of Dox (left) and in the presence of Dox (right).

We isolated fibroblasts, hematopoietic cells and hepatic cells from chimeric mice at different developmental stages (E13.5, newborn, one-week old and adult (four weeks old)), and re-reprogrammed these to generate 2nd miPSCs. Generated iPS colonies were stained with anti-nanog antibody at two weeks after Dox addition and nanog positive colonies were counted and reprogramming efficiency was calculated by dividing the total number of Nanog positive colonies by the number of seeded cells. The reprogramming efficiency of E13.5 fibroblast (MEF), newborn fibroblast (NB fb), one-week old fibroblast (1wk fb) and adult fibroblast (Adult fb) were 5.07%, 3.07%, 1.43% and 0.03%, respectively (Fig. 3B). These results indicate that reprogramming efficiency decreased as developmental stage progressed.

Similar results were observed in reprogramming of hematopoietic cells. Only a few colonies were generated from adult hematopoietic cells; hematopoietic stem cells (HSC), hematopoietic progenitor cells (HPC), myeloid progenitor cells (MP) and macrophages (Mac), when compared with E13.5 CD45+ fetal liver hematopoietic cells (FL CD45) (efficiency was 1.47%) (Fig. 3B). The most remarkable difference was observed in hepatic cells. Fetal liver cells (Fetal hep) were 1200 times more efficiently reprogrammed than adult liver cells (Adult hep) (Fig. 3B). To elucidate whether this difference is attributable to cell division rate, we analyzed the proliferation of fibroblast (MEF, NB fb, 1wk fb and Adult fb) at three, four and five days after seeding with or without Dox. Doubling times were 9.5 hrs, 11.5 hrs, 12.6 hrs and 10.7 hrs, respectively in the absence of Dox; and 27.0 hrs, 21.0 hrs, 29.6 hrs and 25.6 hrs, respectively in the presence of Dox. Although slower proliferation was observed in Dox additive culture, statically no significant differences were observed among MEF, NB FB, 1wk FB and Adult FB (Fig. 3C). Furthermore, we compared the expression levels of reprogramming factors (*Oct4*, *Klf4*, *Sox2* and *c-Myc*) and senescence-related genes (*p19^Arf^*, *p16^INK4a^*, *p53* and *p21^CIP1^*) in four fibroblasts cultured in the absence of Dox by RT-PCR. However, there were no significant differences in the expression of the reprogramming factors (Fig. S2A) nor in the age dependent increase in senescence-related genes (Fig. S2B).

These results indicate that aging effects other than cell proliferation or expression levels of reprogramming factors or senescence-related genes reduced the reprogramming efficiency.

### Effect of c-Myc Expression on Reprogramming

**Figure 4:**
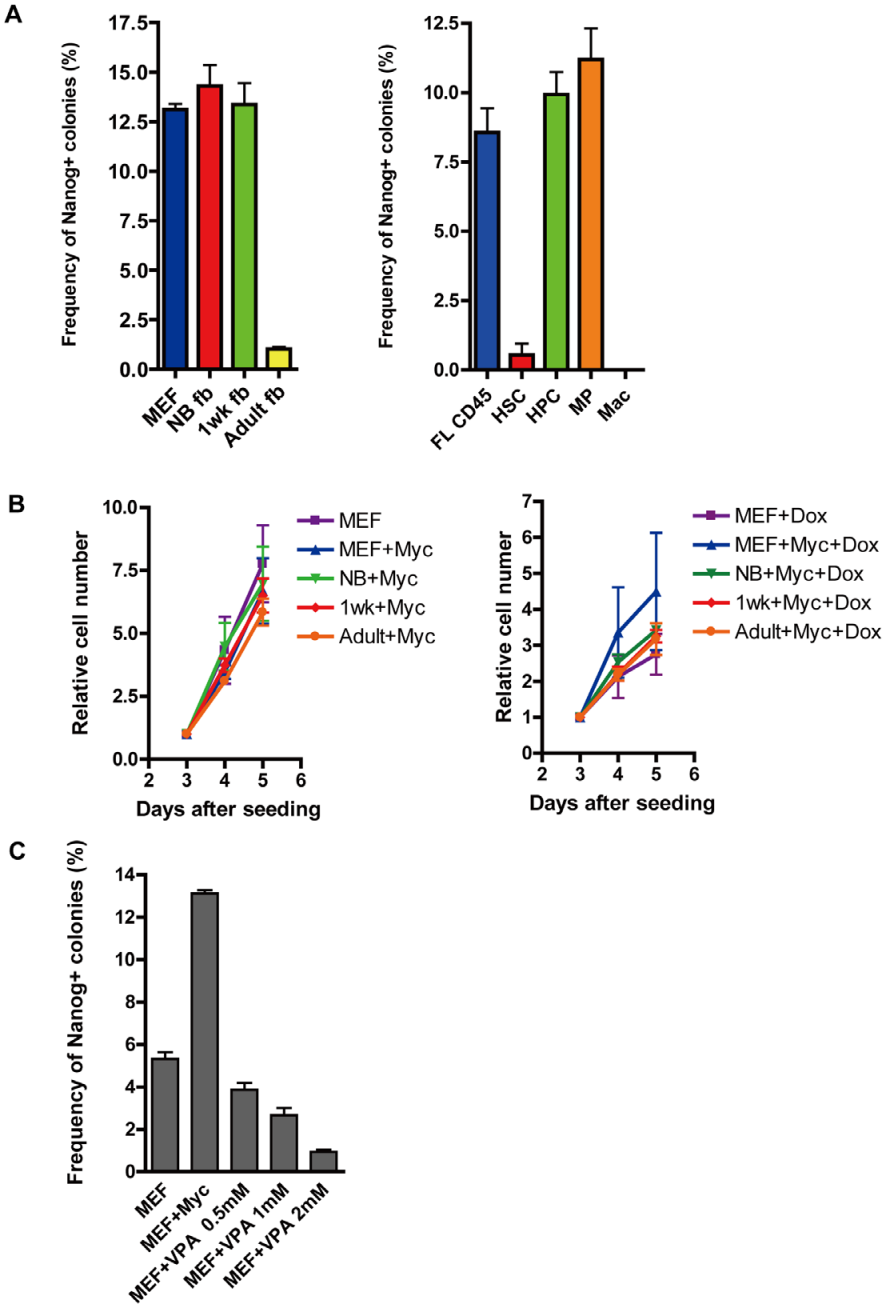
Reprogramming efficiency of somatic cells in the presence of c-Myc. (A) Reprogramming efficiency of fibroblasts (MEF, NB fb, 1wk fb and Adult fb), hematopoietic cells (FL CD45, HSC, HPC, MP and Mac) by four reprogramming factors including *c-Myc*. (B) Cell proliferation rate of fibroblasts (MEF, NB fb, 1wk fb and Adult fb) at three, four and five days after seeded in the absence of Dox (left) and in the presence of Dox (right). (C) Reprogramming efficiency of MEF were compared between reprogrammed by three factors (Oct4, Klf4 and Sox2), four factors (*Oct4*, *Klf4*, *Sox2* and *c-Myc*) and three factors plus VPA (0.5 mM, 1 mM and 2 mM).

Previous report showed that four transcription factors (*Oct4*, *Klf4*, *Sox*2 and *c-Myc*) can reprogram somatic tissues to pluripotency more efficiently than three factors (*Oct4*,*Klf4* and *Sox2*) [20]. Therefore, to ascertain whether this phenomenon is also observed in our system, we generated chimeric mice by transduction of a c-myc-encoded lentiviral vector into clone #6 (#6M). Then, somatic tissues from the chimeric mice were isolated and each reprogramming efficiency was determined. The efficiencies of four-factor reprogramming were 13.1% for MEF, 14.3% for NB fb, 13.4% for 1wk fb and 1.0% for Adult fb (Fig. 4A). Although the efficiencies of three-factor reprogramming declined as developmental stages progressed, we did not detect any significant differences among MEF, NB fb or 1wk fb by four-factor reprogramming (Fig. 4A). Similarly, the age-dependent decline of reprogramming in hematopoietic cells (FL CD45, HPC and MPC) was improved by *c-Myc* (Fig. 4A). Although lowered reprogramming efficiency (0.53%) was observed in HSC culture at two weeks after Dox addition, the efficiency reached to 22.6% at three weeks, without any significant difference between HPC (34%) (data not shown). Given the oncogenic properties of *c-Myc*, we analyzed the cell proliferation rate of fibroblasts as described above and observed that there are statically no significant differences among fibroblasts isolated from the four-factor chimeric mice (Fig. 4B). Because the previous study showed that histone deacetylase inhibitor, valproic acid (VPA) [26], [27], can substitute for *Myc* in iPSCs generation, we analyzed whether VPA can substitute for *Myc* in our system. However, VPA did not increase the reprogramming efficiency in our system (Fig. 4C). These results indicate that *c-Myc* can cancel the aging effects on reprogramming efficiency in some types of cells and reprogramming recovery by *c-Myc* is not necessarily related to histone acetylation.

### Reprogramming Efficiency of Somatic Tissues from other Species

**Figure 5:**
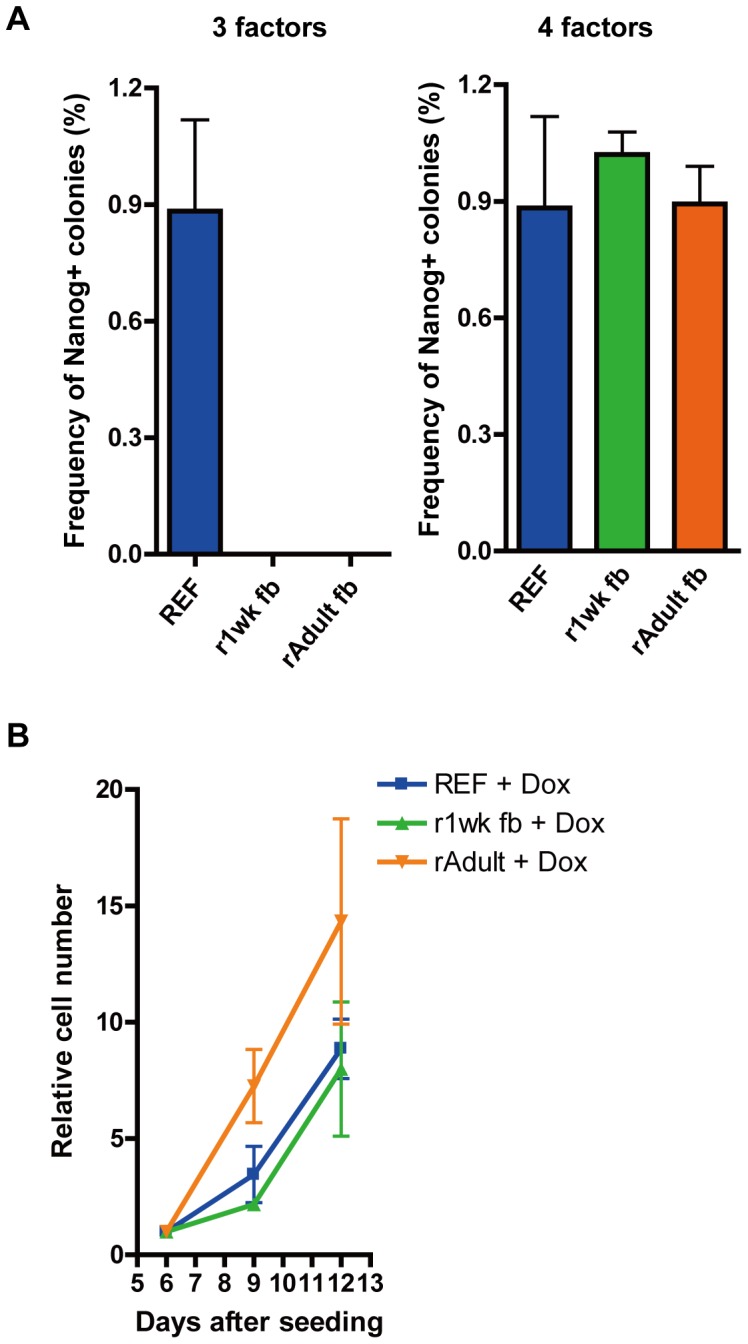
Reprogramming efficiency of rat somatic cells. (A) Reprogramming efficiency of rat fibroblasts (REF, r1wk fb and rAdult fb) by three reprogramming factors (*Oct4*, *Klf4* and *Sox2*) (left) and by four reprogramming factors (*Oct4*, *Klf4*, *Sox2* and *c-Myc*) (right). (B) Cell proliferation rate of rat fibroblasts in the presence of Dox.

We previously reported the successful generation of reprogrammable rat by Ai-LV, and one of the features of our vector system is that it is not limited to an inducible mouse model [28], [29]. By using this reprogrammable rat, we compared the reprogramming efficiency of rat fibroblasts (embryonic fibroblasts (REF), one-week fibroblasts (r1wk fb) and adult fibroblast (rAdult fb)) at 3weeks after seeding. Reprogramming efficiency of REF, r1wk fb and rAdult fb were 0.9%, 0% and 0%, respectively (Fig. 5A). We also analyzed the proliferation of REF, r1wk fb and rAdult fb at six, nine and twelve days after seeding in the presence of Dox and we found that statically no significant differences were observed (Fig. 5B).

These results indicate that the aging-dependent decline of reprogramming efficiency is also seen in rat somatic cells and this is independent of cell proliferation rate. To analyze *c-Myc* functions to initiate reprogramming, we infected REF, r1wk fb and rAdult fb with inducible a lentiviral vector carrying the *c-Myc* gene. The reprogramming efficiencies of these tissues were 0.9%, 1.0% and 1.2%, respectively, indicating that expression of *c-Myc* in addition to *Oct4*, *Sox2* and *Klf4* can cancel the aging effects as seen in mouse somatic cells (Fig. 5A).

## Discussion

We here document a novel vector system which allows Dox-inducible gene expression of 2A linked three reprogramming factors. Unlike the previous system, iPSCs can be induced by a single vector carrying both TRE and rtTA cassettes. In the previous report, only a small percentage of MEF (approximately 0.0084%) was reprogrammed with both the Dox inducible lentiviral vector carrying 2A linked four reprogramming factors (*Oct4*, *Klf4*, *Sox2* and *c-Myc*) and the rtTA vector [12]. On the other hand, in spite of using only three factors, the reprogramming efficiency from Ai-LV infection was approximately twenty times higher than the previous system. This is one of the benefits of a single cassette Ai-LV system. Moreover, it can be more easily constructed with a variety of species. On the other hand, in contrast to previous report we could not generate hiPSCs by Ai-LV in the absence of *hc-MYC* [20]. The possible explanations for this result can be follows; #1; we may require over 5×10^4^ cells for infection. #2; the expression condition for reprogramming factors from Ai-LV may not be optimal [30].

Although we could not generate F1 mice from iPS#6, a certain degree of germ cell contribution was observed in chimeric mice (data not shown). A previous report showed that immature histone acetylation by three reprogramming factors leads to lower efficiency of germline transmission as compared to four reprogramming factors [22]. Since the lowered efficiency was recovered by histone deacetylase inhibitor Tricostatin A (TSA), it is possible to generate F1 mice from iPS#6 by the same treatment. Moreover, because our previous study showed that rat iPSCs derived from Ai-LV infected REF can generate an F1 rat, Ai-LV is considered to have the potential to generate germline competent pluripotent stem cells [28].

As previously described, we found that 2^nd^ iPSCs generated by our reprogramming system posses the functional properties of the original cell type which influences the directed differentiation of iPSCs to their tissue of origin [23], [24], [25]. It should be noted that iPSCs generated by our three factors system exhibit the same profiles as those generated by four factors, including *c-Myc*, suggesting that the epigenetic memory of iPSCs is retained regardless of *c-Myc* status.

There are two major findings in the present study. First, reprogramming efficiency by three factors tends to decrease as developmental stage progressed in several kinds of somatic tissues including fibroblast, hematopoietic cells and hepatic cells. It has been previously reported that up-regulation of senescent effectors *p16^INK4a^*, *p53* and *p21^CIP1^* impairs successful reprogramming [31]. However, we could not detect aging related up-regulation of those effectors among the four kinds of fibroblasts. Moreover, with respect to cell growth, there was no significant difference between embryonic cells (MEF, FL CD45) and aged tissues (NB fb, 1wk fb, Adult fb, HSC, HPC, and MP). Likewise, aged cells did not display characteristic senescence morphology until the iPS colonies started to appear in the embryonic cell culture; indicating that the aging effect other than induction of senescence reduces the efficiency of reprogramming.

Second, addition of *c-Myc* to three factors improved the age related decreases in reprogramming efficiency of mouse and rat somatic cells (NB fb, 1wk fb, r1wk fb, rAdult fb, HSC, HPC, and MP). It has been reported that *myc* increases reprogramming efficiency via chromatin remodeling by recruiting histone acetylase or histone/DNA demethylase [22], [32]. Although histone deacetylase inhibitor VPA could not substitute for *c-Myc* in our system, it is still possible that *c-Myc* may be involved in improving the age related decrease in reprogramming efficiency through histone/DNA demethylation. Moreover, previous reports showed that increased expression of TERT, a catalytic subunit of telomerase is important for reprogramming by maintaining telomere length [33]. On the other hand, in spite of *c-Myc* overexpression, decreased reprogramming efficiency did not recover in Adult fb, mac or Adult hep. These results are similar to previously reported four factors reprogramming systems and may be due to inhibitory mechanisms for which *c-Myc* cannot compensate. For instance, higher expression of the ATP-dependent BAF chromatin-remodeling complex in fetal liver cells compared to adult liver cells leads to higher reprogramming efficiency and this is independent of *c-Myc* expression [34]. Thus, various factors appear to be associated with age-related decrease in reprogramming and our system is valuable for exploration of those factors.

In conclusion, our Ai-LV system revealed the link between aging and reprogramming efficiency and will help us to understand the detailed molecular mechanisms of reprogramming.

## Materials and Methods

### Lentiviral Vector Construction and Preparation

All-in-one inducible lentiviral vector (Ai-LV) was derived from the self-inactivating (SIN) lentiviral vector CS-CDF-CG-PRE [35]. Mouse *Oct4*, *Sox2* and *Klf4* linked by the 2A sequence (BsiWI-EcoRI) was cloned into T7 blue cloning vector (Takara Bio, Shiga, Japan) resulting in T7 mOKS. Polymerase chain reaction (PCR)-amplified TRE fragment (MfeI- BsiWI, EcoRI, NheI, XbaI and XhoI) from pTRE-tight (Clontech Inc. California, USA) was cloned into the EcoRI-XhoI site of CS-CDF-CG-PRE resulting CS-TRE-PRE. PCR-amplified PRE (EcoRI-NheI), human Ubiquitin C (Ubc) promoter (NheI-XbaI), reverse tet tarnsactivator (rtTA) (XbaI-XhoI) from pTet-on Advanced (CLONTECH) and IRES2 EGFP (XhoI-XhoI) from pIRES2-EGFP were cloned into the EcoRI-NheI site, NheI-XbaI site, XbaI-XhoI site and XhoI site of CS-TRE-PRE, respectively, resulting in CS-TRE-PRE-Ubc-tTA-I2G. Then a BsiWI-EcoRI fragment of T7 mOKS was inserted into BsiWI-EcoRI sites of CS-TRE-PRE-Ubc-tTA-I2G resulting in CS-TRE-mOKS-PRE-Ubc-tTA-I2G. For *c-Myc* expression, PCR-amplified *c-Myc* (BsiWI-EcoRI) and Puromycin resistant gene (XbaI-XhoI) were cloned into CS-TRE-mOKS-PRE-Ubc-tTA-I2G resulting in CS-TRE-c-Myc-Ubc-puro. Lentiviral vectors pseudotyped with the vesicular stomatitis virus G glycoprotein were produced as described previously.

### Cell Culture

Mouse embryonic fibroblasts (MEFs) were cultured in Dulbecco’s modified Eagle’s medium (DMEM; Sigma, St. Louis, MO) supplemented with 10% fetal bovine serum (FBS; Hana-Nesco Bio, Moregate BioTech, Australia), 1% L-glutamine penicillin streptomycin (Sigma, St. Louis, MO). Mouse iPS cells (miPSCs) were maintained on mitomycin-c treated mouse embryonic fibroblasts (MEFs) in ES/iPS medium: DMEM supplemented with 15% fetal bovine serum (FBS; Nichirei Bioscience, Tokyo, Japan), 0.1 mM 2-mercaptoethanol (Invitrogen, San Diego, CA, USA), 0.1 mM nonessential amino acids (Invitrogen), 1 mM sodium pyruvate (Invitrogen), 1% L-glutamine penicillin streptomycin (Sigma), and 1000 U/ml of mouse leukemia inhibitory factor (LIF; Millipore, Bedford, MA, USA). Rat iPS cells (riPSCs) were maintained as previously described.

### *In vitro* Differentiation of miPSCs

To allow miPSCs to differentiate into EBs, iPSCs were trypsinized and collected in complete EB differentiation medium (EBD) [36]. Cells were transferred into a 100-mm Petri dish at 2×10^5^ cells per 10 ml EBD. The medium was changed on day four of culture and every two days thereafter. On day six, EBs were trypsinized and stained with phycoerythrin-conjugated (PE-) anti-mouse CD41 and allophycocyanin-conjugated (APC-) anti-mouse c-Kit antibodies (BD Biosciences, San Jose, CA) and sorted CD41^+^,c-Kit^+^ cells on OP9 cells. OP9 cells were maintained in α-MEM containing 15% FCS. 10^5^ OP9 cells were plated in each well of a 6-well tissue culture plate two days before starting co-culture. Co-cultures were employed with IMDM containing 20 ng/ml mouse stem cell factor (SCF) and 20 ng/ml human thrombopoietin (TPO) (PeproTech, Rocky Hill, NJ), 10% FCS, 2 mM L-Gln, 0.1 mM 2-ME, and 100 U/ml penicillin/streptomycin. On day four of co-culture, cells were recovered from the culture dishes for analysis on a flow cytometer.

### Generation of Primary iPSCs and Chimeric Mice

To establish miPSCs MEFs were transduced with Ai-LV and cultured in the presence of Dox (2 ug/ml). Eight days later, generated colonies were picked up and mechanically dissociated cells were placed on MEFs feeder. Chimeric mice were generated by injection of primary miPSCs into day 4.5 blastocysts of ICR female mice, followed by transfer into host uteri as previously described [29]. All of the studies were derived from independent founder animals.

### Isolation of Somatic Cells

Mouse and rat fibroblasts including MEF, REF, NB fb, 1wk fb, r1wk fb, Adult fb and rAdult fb were isolated from E13.5 mouse embryo, E14.5 rat embryo, new born mice, one-week-old mice, one-week-old rats, four-week-old mice and four-week-old rats, respectively. GFP+ mouse fibroblasts were sorted on a feeder layer by a MoFlo™ flow cytometer. For isolation of FL CD45+ cells, E13.5 fetal liver cells were stained with allophycocyanin (APC)-conjugated anti-mouse CD45 (BD Biosciences) and sorted on a feeder layer by a MoFlo™ flow cytometer. For isolation of Hematopoietic stem cells (HSCs) (CD34^−^,c-Kit^+^, Sca1^+^,lin^−^), Hematopoietic progenitor cells (HPCs) (CD34^+^, c-Kit^+^, Sca1^+^,lin^−^) and Myeloid progenitor (MP) (c-Kit^+^, Sca1^−^, lin^−^), Bone marrow (BM)cells from four-week-old mice were stained with an antibody mixture consisting of anti-mouse biotinylated anti-Gr-1, anti-Mac-1, anti-CD45R, anti-CD4, anti-CD8, anti-IL-7R, and anti-TER119 antibodies (eBioscience). Lineage^+^ cells were then depleted using MACS anti-biotin microbeads and a LS-MACS system (Miltenyi Biotec, Bergisch Gladbach, Germany). The cells were further stained with anti-mouse Alexa Fluor 700-conjugated anti-CD34, Pacific Blue-conjugated anti-Sca-1, and APC-conjugated anti-CD117 antibodies, as well as with APC-Cy7-conjugated streptavidin antibody for biotinylated antibodies (eBioscience). For isolation of Macrophage, BM cells from four-week-old mice were stained with an APC-conjugated anti-mouse Mac-1 (BD Biosciences) antibody. Sorting was performed on a FACSAria (Becton Dickinson, Franklin Lakes, NJ). Fetal liver cells (CD45^−^Ter119^−^c-Kit^−^Dlk^+^CD133^+^) were prepared from E13.5 mice. Minced fetal liver tissues were dissociated with 0.05% collagenase solution and were isolated by a MoFlo™ flow cytometer. Adult hepatocytes and non-parenchymal cells were isolated from postnatal livers following a 2-step collagenase digestion. Perfused liver tissues were subsequently dissociated with 0.05% collagenase solution in 10 min at 37°C. The mature-hepatocyte fraction was separated from non-parenchymal cells by several episodes of low-speed centrifugation (50 g, 1 min). Dead cell debris was removed by centrifugation in 50% Percoll solution (GE Healthcare UK, Amersham, UK). CD133+Dlk+ cells or adult hepatocytes were sorted onto feeder cells.

### Induction of 2^nd^ iPSCs

All isolated somatic cells were cultured in the presence of Dox (2 ug/ml) for induction of 2^nd^ iPSCs. Fibroblasts and hematopoietic cells were cultured in ES/iPSC medium. FLCD45 were cultured in the presence of 10 ng/ml human TPO, 10 ng/ml mouse EPO, 10 ng/ml mouse IL-3, 10 ng/ml mouse IL-6, 10 ng/ml mouse Flt3 ligand, 10 ng/ml mouse GM-CSF, 10 ng/ml mouse VEGF and 50 ng/ml mouse SCF (Peprotech). HSCs, HPCs and MPs were cultured in the presence of 10 ng/ml human TPO, 10 ng/ml mouse IL-3, 10 ng/ml mouse IL-6 and 10 ng/ml mouse Flt3 ligand (Peprotech). Macrophages were cultured in the presence of 5 ng/ml M-CSF (Peprotech).

Liver cells were cultured in a 1∶1 mixture of H-CFU-C medium (DMEM/F-12 with 10% FBS or 10% KSR, 1x Insulin-Transferrin-Selenium X, 10 mM nicotinamide, 10-7 M dexamethasone, 2.5 mM HEPES, 1x penicillin/streptomycin/L-glutamine and 1x non-essential amino acid solution and fresh DMEM/10% FBS).

### Alkaline Phosphatase (ALP) Staining and Immunostaining

Alkaline phosphatase (ALP) staining was performed with Vector Red Alkaline Phosphatase Substrate Kit I (Vector Laboratories, Burlingame, CA) according to the manufacturer’s instructions. 5×10^4^ MEFs were infected by Ai-LV at m.o.i 0.4 and ALP positive colonies were counted 14days after infection in triplicate cultures. Efficiency of ALP positive colonies were calculated by dividing transduced cell number (2×10^4^) by the number of ALP positive colonies.

Immunostaining assays were performed as follows. Cells were fixed in 4% paraformaldehyde for 10 min and washed twice with PBS. The fixed cells were incubated in MAXblocking medium (Active Motif, Carlsbad, CA) for 30 min at room temperature (RT) for blocking. The cells were then incubated with primary antibody overnight at 4°C. The day after, cells were washed with PBS and incubated with secondary antibody in PBS for 30 min at RT. Thereafter the cells were washed with PBS and 4′,6 -diamidino-2-phenylindole (DAPI) was added for nuclear staining. For iPS colony count, we performed an enzyme antibody technique by staining the cells with diaminobenzidin (DAB) solution (0.05% DAB, 50 mM Tris/HCI pH 7.4, 0.01% H_2_0_2_ freshly prepared) at 2weeks after seeding for miPSCs or 3weeks after seeding for riPSCs. Primary antibody used was rabbit anti-mouse Nanog antibody (ReproCELL, Kanagawa, Japan, 1∶100). Secondary antibodies were Alexa Fluor 546 conjugated goat anti-rabbit IgG antibody or horseradish peroxidase (HRP) conjugated goat anti-rabbit IgG antibody (Invitrogen, Carlsbad, CA, 1∶300).

### RT-PCR and Quantitative PCR

Total RNA was isolated using RNAeasy kit (Qiagen, Valencia, CA) followed by cDNA synthesis using super script III reverse transcriptase (Invitrogen). PCR was performed using EX Taq HS (Takara) under the following conditions: 94°C for 1 min, followed by 30 or 35 cycles of 94°C for 30 sec, annealing temperature (from 50°C to 62°C) for 30 sec and 72°C for 30 sec, with a final extension at 72°C for 7 min. Quantitative PCR was performed using FastStart Universal SYBR Master (Roche Diagnostics, Germany) for reprogramming factors expression and iPS Efficiency Check qPCR Kit (TAKARA) for aging related gene expression. The primer sequences are listed in Table S1.

All experiments were performed under institutional guidelines.

Animal experiments were performed with approval of the Institutional Animal Care and Use Committee of the Institute of Medical Science, University of Tokyo (permit numbers: A09–29, A10–23, PA11–69).

## Supporting Information

Figure S1 **Phenotypic analysis of mouse and human iPSCs.** (A) iPS#6 and #19 clones was differentiated by removal of MEF and Lif for two weeks and re-reprogrammed by addition of Dox. (Left; in the absence of Dox right; in the presence of Dox) (B) Proviral copy number of isolated iPS clones was analyzed by Southern blot analysis. (C) Karyotype analysis of iPS#6 clone. (D) AP staining of human iPS clone (left). Reprogramming analysis of in vitro differentiated human iPS cells in the absence of Dox (middle) and in the presence of Dox (right).
(TIF)

Figure S2 **Expression profile of reprogramming genes and aging related genes in fibroblasts.** (A) Expression levels of reprogramming genes (*Oct4*, *Klf4*, *Sox2* and *c-Myc*) in fibroblasts (MEF, NB fb, 1wk fb and Adult fb) compared to ESCs. (B) Expression levels of aging related genes (*p53*, *p21^CIP1^*, *p16^INK4a^* and *p19Arf*) in fibroblasts (MEF, NB fb, 1wk fb and Adult fb) compared to ESCs.
(TIF)

Table S1 **The primer sequences for RT-PCR and Quantitative PCR.**
(XLSX)
