# Supplementary figures and images for: Development of an All-in-One Inducible Lentiviral Vector for Gene Specific Analysis of Reprogramming
Source: PLoS One. 2012 Jul 18;7(7):e41007. doi: 10.1371/journal.pone.0041007 (PMC3399796; doi:10.1371/journal.pone.0041007)

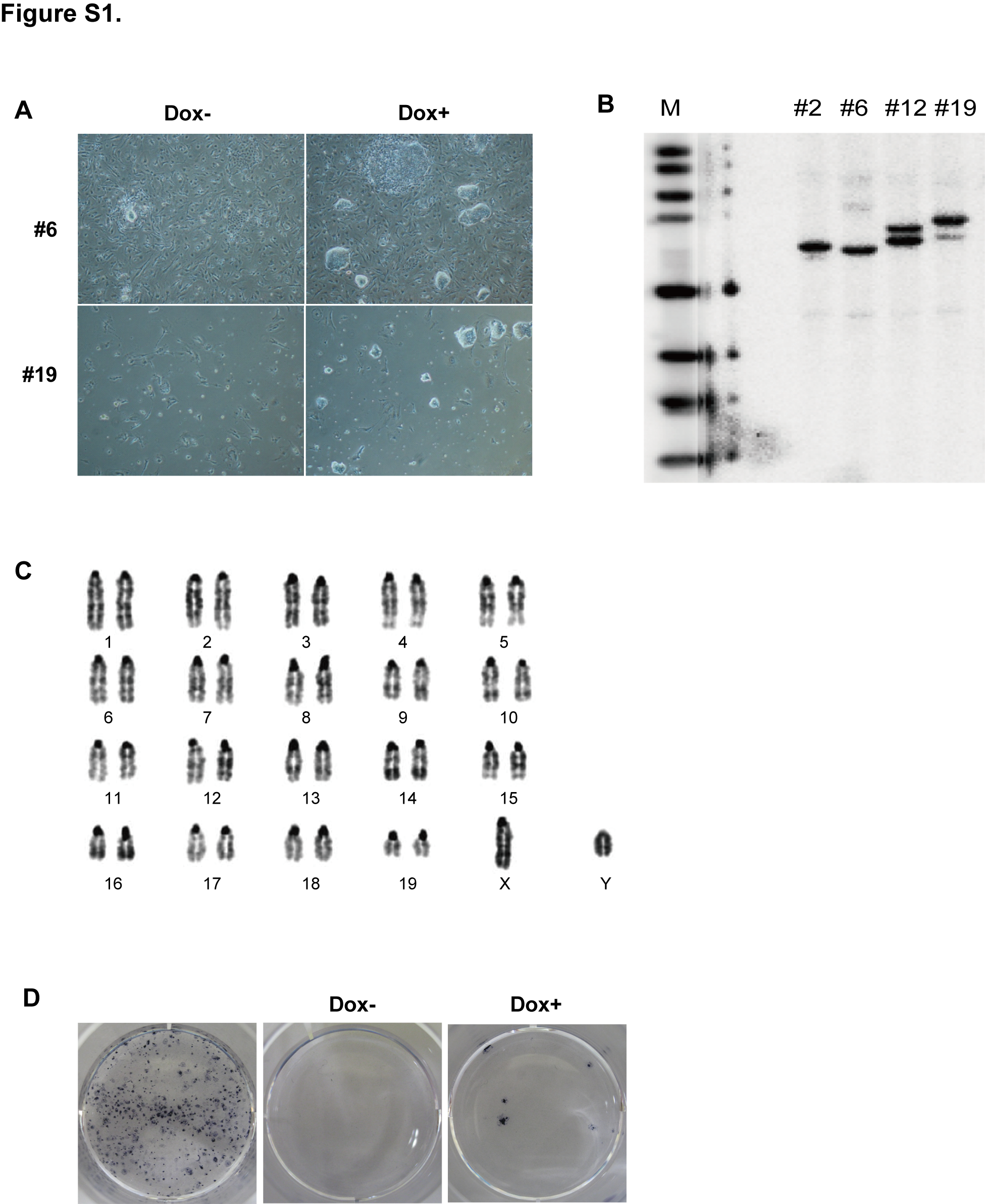

Supplement: Figure S1 — Phenotypic analysis of mouse and human iPSCs. (A) iPS#6 and #19 clones was differentiated by removal of MEF and Lif for two weeks and re-reprogrammed by addition of Dox. (Left; in the absence of Dox right; in the presence of Dox) (B) Proviral copy number of isolated iPS clones was analyzed by Southern blot analysis. (C) Karyotype analysis of iPS#6 clone. (D) AP staining of human iPS clone (left). Reprogramming analysis of in vitro differentiated human iPS cells in the absence of Dox (middle) and in the presence of Dox (right). (TIF) [file pone.0041007.s001.tif]

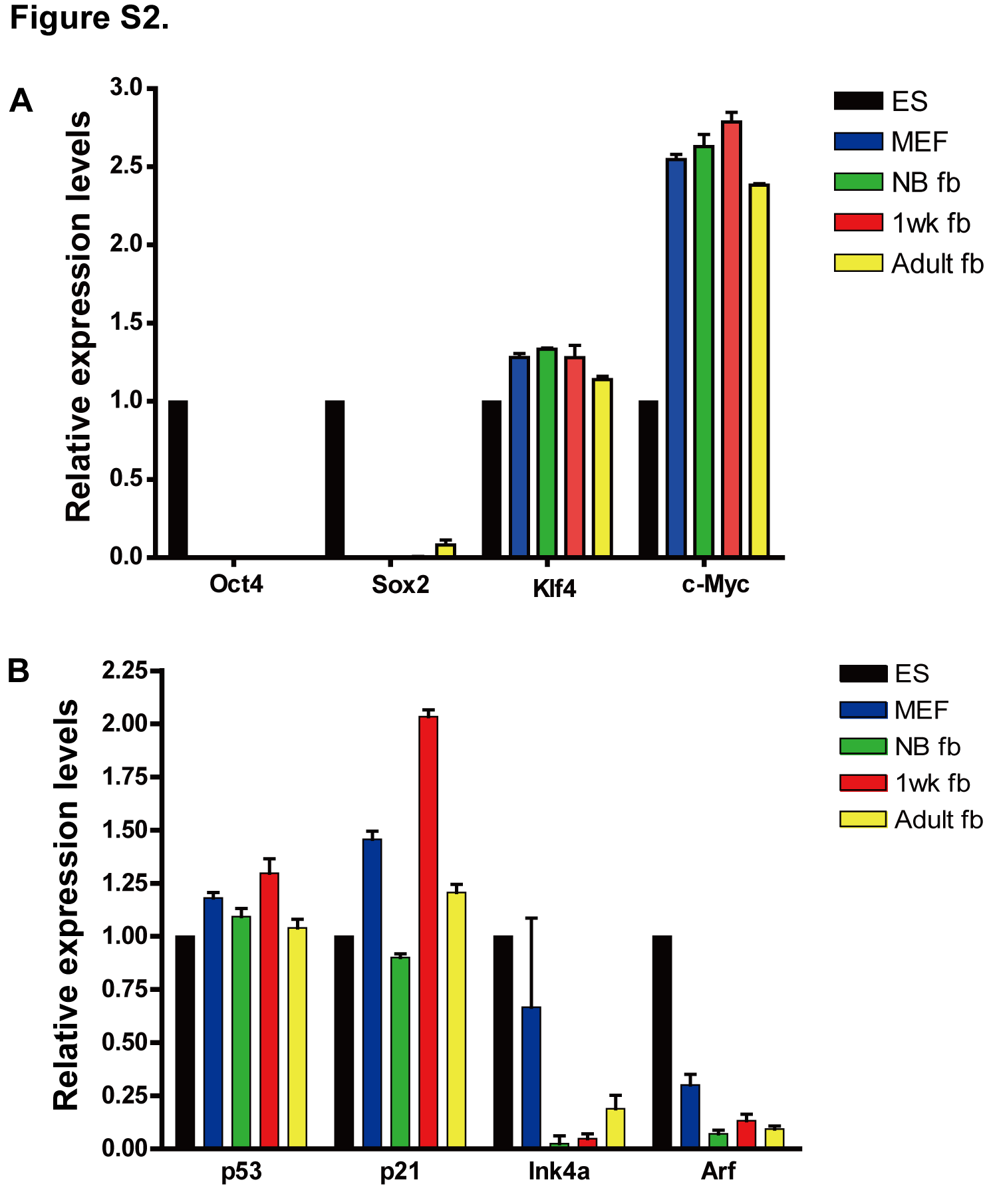

Supplement: Figure S2 — Expression profile of reprogramming genes and aging related genes in fibroblasts. (A) Expression levels of reprogramming genes (Oct4, Klf4, Sox2 and c-Myc) in fibroblasts (MEF, NB fb, 1wk fb and Adult fb) compared to ESCs. (B) Expression levels of aging related genes (p53, p21CIP1, p16INK4a and p19Arf) in fibroblasts (MEF, NB fb, 1wk fb and Adult fb) compared to ESCs. (TIF) [file pone.0041007.s002.tif]
